# Towards the “Baby Connectome”: Mapping the Structural Connectivity of the Newborn Brain

**DOI:** 10.1371/journal.pone.0031029

**Published:** 2012-02-07

**Authors:** Olga Tymofiyeva, Christopher P. Hess, Etay Ziv, Nan Tian, Sonia L. Bonifacio, Patrick S. McQuillen, Donna M. Ferriero, A. James Barkovich, Duan Xu

**Affiliations:** 1 Department of Radiology and Biomedical Imaging, University of California San Francisco, San Francisco, California, United States of America; 2 Department of Pediatrics, University of California San Francisco, San Francisco, California, United States of America; Tel Aviv University, Israel

## Abstract

Defining the structural and functional connectivity of the human brain (the human “connectome”) is a basic challenge in neuroscience. Recently, techniques for noninvasively characterizing structural connectivity networks in the adult brain have been developed using diffusion and high-resolution anatomic MRI. The purpose of this study was to establish a framework for assessing structural connectivity in the newborn brain at any stage of development and to show how network properties can be derived in a clinical cohort of six-month old infants sustaining perinatal hypoxic ischemic encephalopathy (HIE). Two different anatomically unconstrained parcellation schemes were proposed and the resulting network metrics were correlated with neurological outcome at 6 months. Elimination and correction of unreliable data, automated parcellation of the cortical surface, and assembling the large-scale baby connectome allowed an unbiased study of the network properties of the newborn brain using graph theoretic analysis. In the application to infants with HIE, a trend to declining brain network integration and segregation was observed with increasing neuromotor deficit scores.

## Introduction

During brain maturation, structural and functional pathways are formed and reshaped in cases of prenatal, perinatal or early childhood brain injury. Studying these pathways *in vivo* remains a challenge. With advances in MRI, it has become possible over the last decade to noninvasively characterize large white matter bundles using diffusion MRI. The technique has been widely applied to both the adult and the baby brain [1], and has led to new insights into the tissue microstructure of individual tracts. Tractography has been extensively used to visualize white matter tracts and offer tract-based regional analyses. More recently, studies in the adult brain [2]–[4] have attempted to provide a more complete description of the brain's structural connectivity by assembling the “connectome,” a term introduced by Sporns et al. [5] in analogy to the human genome. In these recent studies, the analysis included not only single tracks and regions-of-interest (ROIs) but also the whole brain structural network topology, as assessed at the scale possible using diffusion MRI techniques. The brain network describes interregional mesoscale connectivity patterns of the brain and can be represented by the connectivity matrix (also called “adjacency matrix”) of size *n^2^*, where *n* is the number of brain regions (nodes). Graph theoretic analysis can be applied to the connectivity matrices in order to extract important network characteristics [6], [7]. Key concepts to describe and quantify complex brain networks include local topological parameters, such as node centrality, and global (aggregate) parameters, such as characteristic path length and average clustering coefficient that in concert may indicate the presence of so called “small-world” network characteristics [8]. Studying the human connectome using network science offers a unique opportunity to better understand inter-individual differences in neural connectivity.

The *purpose of this study* was to establish a framework for assessing structural connectivity in the newborn brain at any stage of development, starting with premature neonates, and to show how such a framework could be used to characterize structural network properties in a cohort of six-month old infants with hypoxic ischemic encephalopathy (HIE). Babies with neonatal encephalopathy face a much higher risk of neurological and developmental deficits that are difficult to predict on an individual basis [9]. Characterization of individual structural connectivity networks, together with conventional anatomic MRI imaging, may provide valuable anticipatory information about the potential for encountering abnormalities at a later stage in development. Our hypothesis in this work was that the topological trajectory of the baby brain network is altered by perinatal HIE, and as a result the observed clinical severity of injury would correlate to different structural network phenotypes at 6 months.

Imaging newborn infants poses several unique technical challenges. For reliable structural connectivity network construction and characterization, the following issues had to be addressed:

*data quality assurance*. Data quality suffers from bulk motion, particularly in unsedated infants. Therefore, it is necessary to analyze the occurrence of corrupted diffusion-weighted images and develop an algorithm for their correction or rejection, as in the case of information loss due to motion during half-Fourier acquisition [10].
*automated and unbiased definition of network nodes* of the connectome. Another challenge that had to be addressed for the proposed work was the need for an automated and yet unbiased cortical parcellation scheme suitable for objective evaluation in the developing brain. No single universally accepted parcellation scheme currently exists for human brain regions [5]. In previous studies of the adult human brain [3], [4], [11], [12] parcellation of the brain into nodes was based on anatomic templates and landmarks or functional architecture. Also, a recent study of white matter connectivity in the first years of life [13] used an anatomic template to map the brain at ages of 2 weeks, 1 year, and 2 years. We believe that rapidly changing newborn brains require an unbiased parcellation scheme that does not rely on (adult) brain atlases. This is crucial for the design of both cross-sectional and longitudinal brain imaging studies during the course of development in order to account for the neural plasticity of the pediatric brain. As a part of this work, we propose two different template-free parcellation schemes, and demonstrate their relationship to derived brain network parameters in infants after neonatal HIE.

## Methods

### A. Data Acquisition

All of the MRI scans were compliant with the Health Insurance Portability and Accountability Act (HIPAA) and the study was approved by the Committee on Human Research (CHR) of the University of California, San Francisco. Written informed parental consent was obtained.

As part of a study on neonatal encephalopathy, diffusion tensor imaging (DTI) was performed on 17 six-month old babies who had encephalopathy at birth which affected neurological outcome at 6 months to varying degree. The babies were scanned on a General Electric 3T EXCITE MR scanner using half-Fourier spin-echo (SE) echo planar imaging (EPI) diffusion sequence with a field of view (FOV) of 24 cm×24 cm, 72×128 matrix (half-Fourier with 8 overscans) reconstructed to 128×128 and zero-filled to 256×256, TE = 57 ms, TR = 9 s, 30 directions distributed by electrostatic repulsion [14], b-value = 700 s/mm^2^, with a parallel imaging ASSET (Array Spatial Sensitivity Encoding Technique) acceleration factor of 2. Forty-five to fifty consecutive slices of a 2 mm thickness were acquired through the entire brain, aligned axially along a plane between the genu and splenium of the corpus callosum with the interhemispheric fissure vertically in the midline. The scan time for the DTI sequence was approximately four minutes. Total time for each examination, which also included T_1_-weighted, T_2_-weighted, and spectroscopic imaging sequences, was approximately one hour. The patients were scanned in an 8-channel adult head coil while under anesthesia.

**Figure 1:**
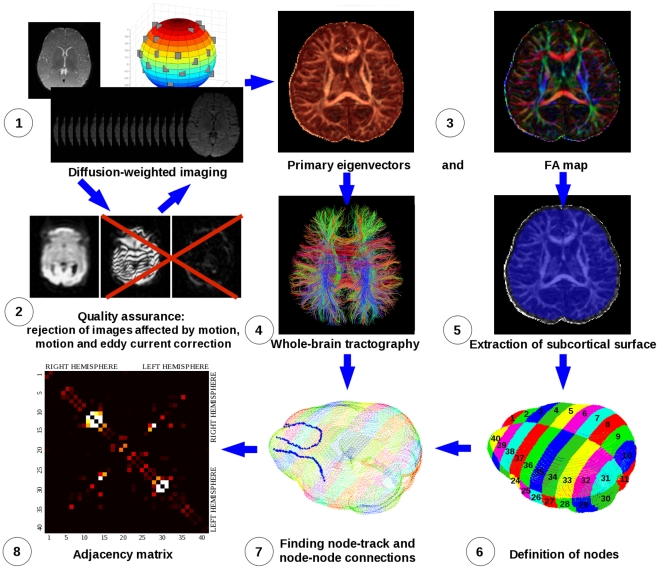
Flowchart: Assembling a Baby Brain Structural Network. After a set of diffusion-weighted images is acquired (1), a quality assurance step is performed in which data affected by motion are rejected and the remaining images are corrected for eddy current distortions and affine head motion (2). Although this step may not be necessary in cooperative adults, it is essential for high-quality tractography in infants. The diffusion tensor is calculated for the resulting data (3), and whole-brain streamline fiber tractography is undertaken (4). The subcortical surface is extracted (5) and partitioned into nodes using either the gridded or equipartition parcellation scheme (6, see below). Node-track and node-node connections are derived (7) and the adjacency matrix is constructed (8).

Data were processed offline and used to construct structural networks according to the following computational pipeline we devised using Matlab, FSL [15], and Diffusion Toolkit [16]. A flowchart depicting the work flow is shown in Fig. 1.

### B. Data Quality Assurance

Data quality often suffers from bulk motion in unsedated babies and due to infant movement despite sedation, in addition to large eddy current artifacts. In diffusion-weighted imaging, problems associated with patient motion are amplified due to the presence of the diffusion-sensitizing gradient pulses [17]. Fast imaging techniques, such as half-Fourier EPI used in our study, help to reduce the scan time and thus the probability of motion during the sequence. On the other hand, the half-Fourier technique increases the sensitivity of the scan to bulk motion. Intra-scan rotation of the imaged object during the application of the diffusion gradient introduces a linear phase shift across the object, which displaces the echo in k-space orthogonal to the direction of the applied diffusion gradient [17]. With half-Fourier imaging, sufficiently high rotational speed may displace the DC component of the k-space outside the sampling range [10]. This results in a dramatic intensity loss in the image that cannot be corrected. Displacement of the DC component in the opposite direction cannot be tolerated either, as the standard homodyne reconstruction produces spurious high-frequency image intensity oscillations.

To insure data quality, we implemented an automated data rejection algorithm to identify and discard directionally-encoded diffusion measurements that are corrupted by motion. Images with intensity ripples or signal loss due to the displacement of the k-space center in half-Fourier imaging were identified as outliers. This was done by pixel-wise analysis of the diffusion-weighted DICOM images. When a certain number of pixels deviated from the corresponding mean pixel value for all diffusion directions by three standard deviations, the direction was not included in the tensor calculation. The threshold for the number of pixels was set empirically, depending on the head size. Slices covering nasal cavities and affected by susceptibility artifacts were excluded from the rejection process.

The remaining DICOM images were converted to the Neuroimaging Informatics Technology Initiative (NIfTI-1) format and corrected for eddy current distortions and simple head motion using affine registration to a reference volume [15].

### C. Data Reconstruction and Tractography

After quality assurance steps were taken, tensor-based reconstruction and whole-brain streamline fiber tractography was performed using Diffusion Toolkit [16]. The deterministic Fiber Assignment by Continuous Tracking (FACT) algorithm was applied [18] using the entire diffusion-weighted volume as the mask image. A threshold angle of 35° was chosen as a compromise between false positive and false negative streamlines [19].

### D. Brain Cortex Extraction, Parcellation into Nodes, and Structural Network Construction

The algorithm for assembling the structural network included subcortical surface extraction, surface parcellation, identification of white matter tracts connecting individual parcellated nodes, and finally, assembly of the connectivity matrix. Subcortical surface extraction was based on the non-zero fractional anisotropy (FA) map. Morphological operations were applied and the surface 2–4 mm below the cortex was extracted, achieving similar results to previously proposed methods [4].

**Figure 2:**
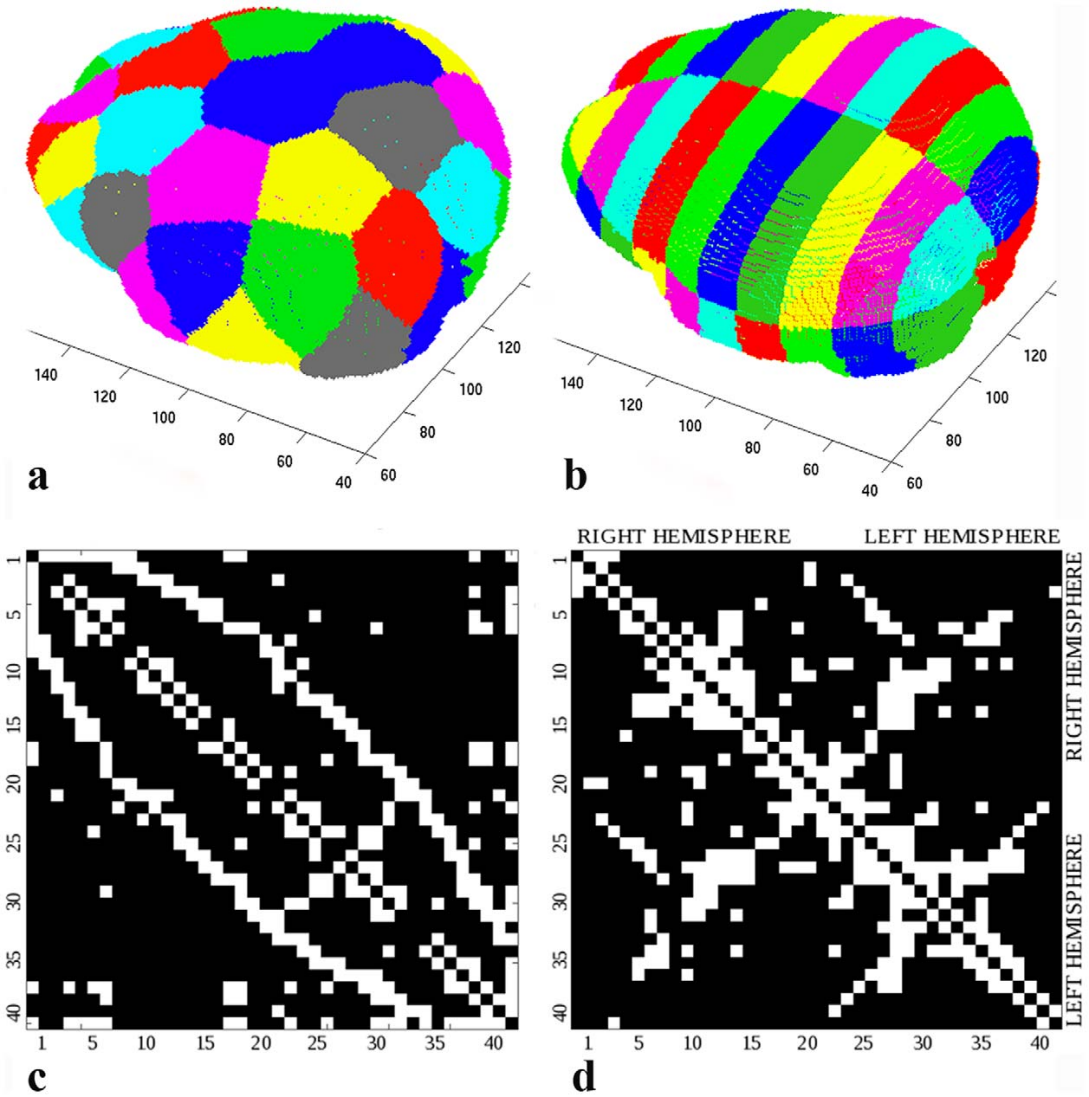
Parcellation Schemes and Adjacency Matrices. a) Equipartition and b) gridded parcellation of the six-month old baby brain. c), d) Adjacency matrices binarized with threshold 1 for both parcellation schemes in a representative baby with NMS 0, for which no diffusion directions were discarded.

Template registration, which has been used to define anatomic nodes in prior work in adult brain [2], [4], is not directly applicable to the developing infant brain which is known to undergo considerable changes in both structure and function. In this work, we developed two unbiased automated methods for parcellating the brain surface:

- the derived subcortical surface was divided into nodes based on Recursive Zonal Equal Area Sphere Partitioning [20] (“***equipartition***”) (Fig. 2 a);
- the brain was partitioned into spatial regions of equal spatial extent along the *x*, *y*, and *z* axes of the imaging volume (“***gridded***”) (Fig. 2 b).

Based on the spatial prescription of the images with the corpus callosum and interhemispheric fissure, the *x*, *y*, and *z* axes of the imaging volume corresponded to the biological developmental axes. Therefore, the gridded parcellation was performed between ventral and dorsal, rostral and caudal, and medial and lateral surfaces of the brain. The equipartition, on the other hand, was not aligned with the anatomy. A unit sphere was first divided into regions of equal area and the set of center points of the regions was determined, in order to serve as the node reference points. The sphere was then scaled to the brain surface and every point on the brain surface was assigned to the closest node reference point. This simple and practical approach resulted in nodes of a similar size.

Note that both approaches avoid imposing arbitrary anatomical constraints on connectivity and therefore may be better suited to address the dynamic structure of the rapidly changing developing brain, in which the sulci and gyri of the adult brain cannot be used as reliable fiducials.

In both cases the number of nodes was chosen to be 40. Previous studies of adult connectivity networks have used between five [11] and 998 [12] cortical regions of interest. For example, Hagmann et al. [21] used a “low-resolution” parcellation into 66 cortical regions of varying sizes using an automated-landmark based algorithm and a “high-resolution” parcellation with 241 ROIs of approximately equal area of 6 cm^2^. In our study, we used 40 ROIs of similar surface area, such that parcellation of the relatively smaller infant brain also resulted in ROIs with surface areas of approximately 6 cm^2^ for babies at a mean gestational age of 31 weeks [22].

Connectivity was then defined using the results of whole-brain fiber tractography. A lower cutoff fiber length of 10 mm was applied in order to remove extremely short tracts from further analysis. Any two nodes were considered to be connected if tracks with two end points located in their respective ROIs were present. Two ROIs may have multiple connections which we can represent by weights on the edges of the network. However, given the inherent noise in the diffusion data and the arbitrary units for edge weights, we instead treated all edges as unweighted. The matrices for all babies were binarized using a threshold in the range from 1 (only one fiber track is required to consider two nodes connected) to 10. The thresholds higher than 1 were used to eliminate pseudoconnections that may come about as the result of noise or modeling error.

### E. Network Graph Analysis

We modeled our connectivity networks as a graph [23], defined as a set of nodes or brain regions, *{N = 1…n}* connected by a set of edges or tracts, *{E = (N_i_,N_j_)…e}*. The graph can be represented by an adjacency matrix, *A*, where *A_ij_ = w* if the *i*th and *j*th nodes are connected by an edge with weight *w*. Our graphs were unweighted and undirected as diffusion MRI provides no information about directionality of the connections. From the adjacency matrix, any network measure can be quantified. As proof of principle we limited ourselves to properties which have been previously reported in adult brains including global measures of segregation (average clustering coefficient, *C*) and integration (characteristic path length, *L*), that together describe the small-world properties of the network.

We define the clustering coefficient of the network as
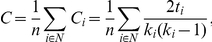
where *k_i_* is the degree, *C_i_* is the clustering coefficient of node *i* (*C_i_* = 0 for *k_i_*<2), and *t_i_* is the number of triangles around node *i* [6].

We define the characteristic path length of the network as
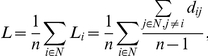
where *L_i_* is the average shortest distance between node *i* and all other nodes and *d_ij_* is the shortest distance between nodes *i* and *j* [6].

Adjacency matrices were constructed for all babies and then analyzed using the Brain Connectivity Toolbox in Matlab [6].

### F. Correlation with the Neurological Outcomes

Neurological outcome was assessed at 6 months by pediatric neurologists blinded to neonatal course, MR imaging findings, and derived structural network parameters. A validated neuromotor score (NMS) was assigned during a standardized neurologic examination: 0, normal; 1, abnormal tone or reflexes; 2, abnormal tone and reflexes; 3, functional deficit of power in addition to tone or reflex abnormality; 4, cranial nerve involvement with motor abnormality; 5, spastic quadriparesis [24]. To assess the relationship between the small-world network properties in babies with observed neurological outcome, linear regression analysis was performed on the derived network metrics (average clustering coefficient and characteristic path length) using Matlab. A *p* value of less than 0.05 was considered statistically significant.

## Results

### Data Quality Assurance

As the overarching aim of this work was to develop a framework suitable for characterization of structural brain networks in babies at any stage of development, the performance of our quality assurance algorithm was demonstrated on the diffusion data acquired for unsedated neonates obtained as a part of an ongoing study of brain injury in newborn babies. Figure 1, step 2 shows two common examples of corrupted diffusion-weighted images acquired on a preterm baby. As described above, the use of half-Fourier k-space sampling reduces the scan time but simultaneously increases the sensitivity of the sequence to rigid-body motion. Depending on the direction of object motion, the DC component of the k-space is displaced either into the high spatial frequency range, causing ripple-like intensity oscillations across the image, or outside of the sampled range of spatial frequencies, causing a dramatic signal loss. In the studied cohort of anesthetized six-month old infants, the algorithm, on average, resulted in rejection of 0.9 diffusion directions. It should be noted, however, that these babies were preselected from a larger cohort based on visual assessment of artifacts. Artifacts in sedated babies were caused by mechanical vibrations of the MRI table, as well as movement caused by the mechanical ventilator used during anesthesia. On visual inspection, rejection of diffusion directions in images with artifacts significantly improved fiber tractography.

### Baby Connectome

Figures 2 c and d show binarized (threshold 1) adjacency matrices for both parcellation schemes in a representative baby with NMS 0, for which no diffusion directions were discarded in the quality assurance step. An element *ij* in the adjacency matrix *A* has a value of one if node *i* is connected to node *j*. The diagonal elements of the matrix represent self-connections. Node numbers were arbitrarily assigned. In the case of the more structured gridded parcellation scheme, the node numbers from one to forty run first through the right hemisphere and then through the left hemisphere. Therefore, non-zero values in the upper left quadrant show association fibers in the right hemisphere and in the lower right quadrant show fibers in the left hemisphere. The two remaining quadrants show commissural fibers connecting two hemispheres. The pattern in the equipartition matrix, however, merely reflects a circular enumeration of the nodes around the cortical surface.

**Figure 3:**
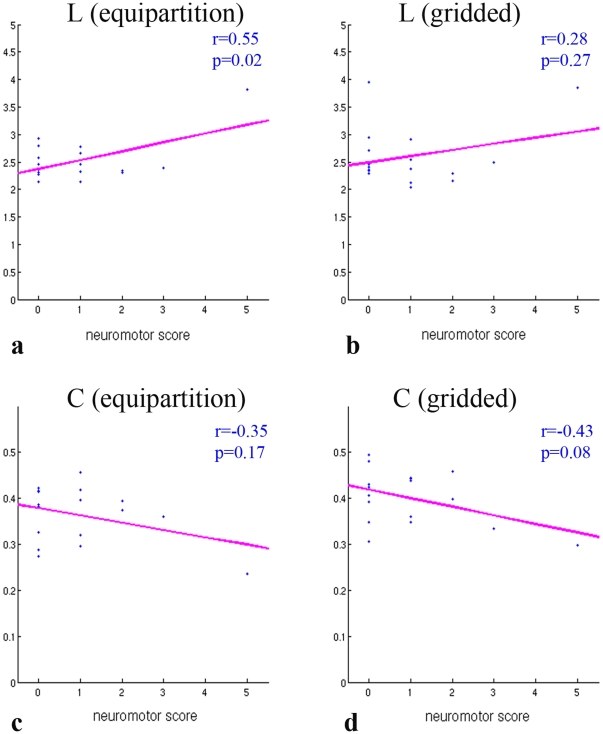
Correlations between Neuromotor Score and Small World Properties. Observed correlations between neuromotor score and characteristic path length (a and b) and average clustering coefficient (c and d) in babies with encephalopathy.

Because the chosen parcellation schemes are not anatomically registered, the resulting adjacency matrices cannot be compared element-wise or multiplied to obtain the skeleton matrix for a group of subjects. Overall network topology, however, can be assessed using aggregate measures such as small-world properties. Characteristic path length *L* showed a positive correlation with NMS for both parcellation schemes (Figs. 3 a and b); however, the correlation was statistically significant for most of the threshold values only for the equipartition parcellation approach. Average clustering coefficient *C*, in contradistinction, decreased with increasing NMS (Figs. 3 c and d). The correlation was significant (*p*<0.05) only for the threshold of 8 with the equipartition scheme. The figures show the results for the threshold of 5. *C* and *L* ranged 0.2–0.5 and 2–4, respectively, for both parcellation schemes across different thresholds (NMS 0). These values are comparable to values reported by several other studies of the structural and functional adult brain networks summarized by Li et al. [25]. The obtained clustering coefficients were above those of random networks (*C*>*C_rand_*), and the characteristic path length was comparable to random networks (*L*∼*L_rand_*). The random networks were obtained by randomizing the binary adjacency matrices, while preserving the degree distribution [6]. This is often referred to as the configuration model [26] and is a standard random model used in the literature to assess statistical significance. Thus, similar to what has been described in adult connectivity networks, the topological structure of the infant network exhibited small-world properties. Small-world networks are neither completely regular nor completely random [8], [27]. Most connections are local, as in regular networks; however, a small number of connections are rewired to reach over a longer distance. This network topology allows for a high efficiency and a high level of adaptation with a very low wiring and energy cost.

The influence of the number of nodes on the network metrics was explored for both parcellation schemes. In the case of the gridded parcellation scheme this was accomplished by varying the number of regions along the rostral-caudal axis. A decrease in the total number of nodes from 40 to 20 resulted in a significant change in the network metrics (a 17–47% increase of the clustering coefficient and a 21–28% decrease of the path length value for both parcellation schemes, NMS 0). However, the 20-node network still demonstrated small-world properties (although it should be noted that the ratio *C/C_rand_* decreased and was lower than 2 in most cases). The trend for the small-world characteristic *L* and *C* depending on NMS remained the same; however, almost none of the thresholds between 1 and 10 resulted in a statistically significant correlation.

## Discussion

In this work, we have developed an automated technique to map structural connectivity in the infant brain using diffusion MRI, and used this approach to characterize large-scale connectivity of the cortex in 17 six-month old babies with HIE at birth. The approach is similar to what has been described in adults, but was modified to include more rigorous quality assurance and an anatomically unconstrained approach to parcellation in order to better study the variable anatomy of this group. Interestingly, the derived networks demonstrated properties which were correlated to neuromotor outcome in HIE babies at six months. The choice of the threshold for binarizing connectivity matrices affected the statistical significance of the resulting correlation. Nevertheless, a trend to declining brain network integration and segregation was observed with increasing neuromotor deficits. These results should be interpreted with caution, as the mechanisms for brain network disruption in babies with encephalopathy have not yet been well-characterized and are likely heterogenous in both etiology and outcome [9]. A larger number of subjects and a longitudinal study design over years would provide more information on this aspect of the study. The principle result that we intended to achieve is the framework for structural network construction in the pediatric brain – a step towards the large-scale baby connectome that will contribute to our understanding of brain development, as well as developmental abnormalities or lesions that affect brain function.

The important step of data quality assurance preceded the mapping of the structural connectivity. This step included considerations for not only inter-image motion and eddy-current correction, but addressed intra-image diffusion encoding artifacts that were not accounted for by traditional motion correction algorithms. Rejection of diffusion directions in half-Fourier images with artifacts significantly improved fiber tractography. Unless the location of the DC component of the k-space is detected and an adaptive version of the homodyne algorithm is used [10], affected images have to be excluded from diffusion data reconstruction. Furthermore, no algorithm can solve the problem if displacement of the k-space center is outside the sampling range.

No single universal parcellation scheme of the brain exists for the infant brain. A major drawback of using anatomical brain regions, such as the Anatomical Automatic Labeling (AAL) atlas, for studying early brain development was pointed out by Fan et al. [28]. Brain networks were built in the same subjects at the ages of 1 month, 1 year, and 2 years based on correlations in regional gray matter volume measures. The authors noted that the AAL atlas might not match very well with function and anatomy of the early development brains. In our study, we proposed two different anatomically unconstrained parcellation schemes. The only anatomic alignment imposed with the proposed gridded parcellation was alignment of the imaging plane with the corpus callosum and interhemispheric fissure, so that the baby brain was evaluated using the same cardinal *x*, *y*, and *z* axes. The equipartition scheme imposed no anatomic constraints. The proposed schemes are straightforward and more suitable for the rapidly changing newborn brain, as they avoid the inherent bias associated with using anatomically-predefined node locations. While the nodes partitioned in the proposed way do not directly correspond to each other across subjects, comparison and assessment are possible using the total resulting network and derived global characteristics. We also expect that, in the future, network-driven co-registration will be an advantageous alternative to atlas-based coregistration, especially in case of challenging age groups and variable anatomy. This will enable unbiased comparison of networks on the local scale, i.e. using single node features. In our study, while both of the parcellation schemes showed the same trend for the small-world metrics, the equipartition demonstrated a stronger correlation with the NMS. The more structured gridded parcellation facilitated visual inspection of the adjacency matrices, in which, e.g. the symmetry of the right and left hemispheres could be easily observed. However, this is only possible in case of a proper alignment of the imaging box with the anatomy. This link to the anatomy, on the other hand, makes the scheme inferior to the equipartition, which is truly automatic and unbiased.

Connections between the cortex and subcortical gray matter structures, such as thalamus, were not analyzed in this work. Including those connections into the analysis would require a relatively precise definition of the inner brain structures manually and/or using templates and, thus, hinder the universal, fully automated approach to studying the developing brain. Results of mapping of connections between thalamus and cortex in the adult brain using DTI [29] and using both DTI and fMRI [11] have been reported, but the influence of these structures on overall connectivity is difficult to define.

The proposed framework can be applied to babies of different ages, including premature newborns, and thereby provides a novel tool for unbiased study of structural maturation of the brain. Previously, developmental trajectories could only be studied by measuring anatomy and analyzing separate DTI tracks using tract- or region-of-interest based analysis. We also expect that, by studying brain network topology in newborns, it will become possible to better understand the process of relocation of specific brain functions as a consequence of brain plasticity. The proposed anatomically unconstrained approach to parcellation followed by network-driven analysis of the connectome should facilitate this task.

Recently Hagmann et al. [21] applied the principles of MR connectomics to explore the contribution of white matter maturation to the development of connectivity between 2 and 18 years. Among other network refinements, they observed a significant increase in node strength and efficiency along with a decrease in clustering. The betweenness centrality of brain regions remained largely unchanged, with the precuneus, posterior cingulate cortex, superior frontal cortex, and superior parietal cortex remaining the hub regions with the highest centrality ranks. Another very recent longitudinal study by Yap et al. [13] explored developmental trends of white matter connectivity in healthy pediatric subjects at ages of 2 weeks, 1 year, and 2 years. The results indicated that the small-world architecture exists at birth with efficiency that increases in later stages of development. The framework developed here specifically aims at facilitating similar studies by ensuring the diffusion data quality and anatomically unbiased parcellation in children under the age of 2.

Our graph theoretical analysis showed small-world properties in six-month old babies. The results are in agreement with previous studies of the adult human brain using EEG, MEG, diffusion MRI, and functional MRI (see [30] for a review), as well as with the pediatric studies mentioned in the previous paragraph. While the detection of small-world attributes is considered to be largely independent of the parcellation scheme and spatial resolution [27], [31], the specific network metrics can be affected by both, the network resolution (number and size of nodes), and the angular and spatial resolution of the diffusion acquisition [32]. Zalesky et al. [33] and Hagmann et al. [12] recently showed that the parcellation scale strongly influences the network metrics. We have observed this effect when decreasing the number of nodes from 40 to 20. However, it is also reported that this strong dependence does not suggest that any given parcellation scale is more optimal than another and its choice remains a subject of research.

The patterns of structural connectivity that have been observed in the human brain parallel similar findings of functional connectivity using BOLD fMRI ([11], [21], [34] and [35] for a review of earlier studies). Though interrelated, these two approaches are complementary, and the full description of both structural and functional connectivity is crucial in understanding normal and abnormal maturation of the brain as a whole. Functional connectivity of the newborn brain was studied recently by Fransson et al. [36]. It was shown that at the time of birth, the functional brain connectome largely involves brain regions responsible for sensation and action, whereas only weak involvement was found for heteromodal brain areas. The strong candidates for cortical hubs were found in motor, sensory, auditory, and visual primary cortex. Another study in preterm infants [37] concluded that all resting state networks, including visual, auditory, somatosensory, motor, default mode, frontoparietal, and executive control networks, are present by term. A recent review by Smyser et al. summarizes exploration of the functional organization of the developing brain [38]. However, the importance of the structural network cannot be overemphasized, as functional connections represent a single brain state that unfolds within a milieu of fixed anatomic connections. The combined use of noninvasive structural and functional imaging methods in the same subject would offer the most robust path toward defining the full large-scale connectome. To date, this has been done only for the adult brain, with the structural connectome being the challenging task in the immature brain. The framework developed in this study will facilitate this important step of going from structure to function, which is essential for understanding how cognitive processes emerge from their morphological substrates [11].

In the present study, we used diffusion tensor MRI in combination with deterministic tractography to track white matter pathways. Though fast and straightforward, deterministic tractography produces reliable results only in brain areas where anisotropy is high [29]. As fibers approach the cortex, diffusion anisotropy diminishes, and calculated principal diffusion directions become increasingly uncertain as a result [39]. This has limited attempts to trace pathways directly from deep gray matter, which typically has low anisotropy. To reduce the effect of this limitation, we restrained connectivity mapping to the white matter by choosing the nodes on the subcortical surface 2–4 mm below the cortex. Probabilistic tractography could be used instead to improve fiber tracking. Yo et al. showed that probabilistic approaches show on average more connected regions but lower connectivity values than deterministic methods [40]. High angular resolution diffusion models may also reveal connections between more brain areas than the simple tensor model, by resolving crossing fibers. These differences should be taken into account when comparing results obtained with different frameworks for assembling the connectome.
